# Illusions of control without delusions of grandeur

**DOI:** 10.1016/j.cognition.2020.104429

**Published:** 2020-12

**Authors:** Daniel Yon, Carl Bunce, Clare Press

**Affiliations:** a Department of Psychology, Goldsmiths, University of London, UK; b Department of Psychological Sciences, Birkbeck, University of London, UK

**Keywords:** Agency, Illusions, Action, Perception

## Abstract

We frequently experience feelings of agency over events we do not objectively influence – so-called ‘illusions of control’. These illusions have prompted widespread claims that we can be insensitive to objective relationships between actions and outcomes, and instead rely on grandiose beliefs about our abilities. However, these illusory biases could instead arise if we are highly sensitive to action-outcome correlations, but attribute agency when such correlations emerge simply by chance. We motion-tracked participants while they made agency judgements about a cursor that could be yoked to their actions or follow an independent trajectory. A combination of signal detection analysis, reverse correlation methods and computational modelling indeed demonstrated that ‘illusions’ of control could emerge solely from sensitivity to spurious action-outcome correlations. Counterintuitively, this suggests that illusions of control could arise because agents have excellent insight into the relationships between actions and outcomes in a world where causal relationships are not perfectly deterministic.

## 1 Introduction

False beliefs about action are a common feature of mental illness. Gambling addicts often believe that they can affect random games of chance (Clark, 2010; Gadboury & Ladouceur, 1989), and patients in the throes of psychosis can develop grandiose delusions about the influence they have over the external world (Knowles et al., 2011; Smith et al., 2005). However, decades of research in the cognitive sciences has revealed that healthy individuals also frequently experience ‘illusions of control’ over events that they do not objectively influence (Langer, 1975). For example, we tend not to realise when we push ‘placebo buttons’ attached to pre-programmed traffic crossings, elevators and office thermostats (Luo, 2004), which may reflect exaggerated expectations about control (Moore, 2016). Moreover, classic studies have demonstrated that participants reliably over-report being in control of a flashing lightbulb, even when the flashes are programmed to occur randomly (Alloy & Abramson, 1979; see also Matute et al., 2019; Vázquez, 1987; Yarritu et al., 2014), and recent work suggests agents often believe their actions can stabilise objectively volatile environments they are interacting with (Weiss et al., 2019).

Such illusions have prompted widespread claims that agents are often insensitive to the objective contingencies between actions and outcomes, and instead rely on grandiose beliefs about the extent to which they influence the outside world. While nonveridical, such grandiose beliefs are argued to be adaptive in a number of ways. For example, exaggerated beliefs about agency may be an important feature of healthy self-esteem (Bandura, 2002) – a point underscored by the fact that illusions of control are attenuated in depression (Alloy & Abramson, 1979; Presson & Benassi, 2003; Vázquez, 1987). At the same time, evolutionary simulations have suggested that creatures with unrealistically optimistic beliefs about their actions may enjoy a fitness advantage over populations with unbiased insight (Johnson & Fowler, 2011), particularly in environments where overestimations are not particularly costly (Haselton & Nettle, 2006). Bayesian models of learning and control have also suggested that agents possessing pessimistic beliefs about the controllability of the world miss out on rewards they could have reaped, but those with grandiose beliefs do not suffer (Huys & Dayan, 2009).

However, there is also widespread evidence that humans can be highly sensitive to the objective contingencies between actions and their consequences. For example, explicit judgements of causality (Dickinson et al., 1984) and agency (Sato, 2009), along with implicit markers of action-outcome learning (Elsner & Hommel, 2004), increase as contingencies between actions and events become stronger. Similar patterns are seen in ‘intentional binding’ experiments, where shifts in subjective time perception are used as an implicit marker of the sense of agency (Haggard et al., 2002; Moore et al., 2009; Moore & Haggard, 2008). Against this backdrop, we suggest a potential alternative explanation for illusions of control – that these experiences arise because agents are *especially* sensitive to relationships between actions and outcomes and pick up on those that occur purely by chance. In naturalistic environments, action-outcome correlations are frequently imperfect even when we are in control. For instance, our actions may truly affect the behaviour of a temperamental friend, but these effects may not be perfectly predictable. In experimental settings, although experimenters can know exactly whether a participant is controlling an event, participants themselves can usually only base these decisions on experienced correlations. As such, a sensitive observer might detect correlations in random noise that spuriously resemble situations of control, and therefore experience feelings of agency.

A key difference between this sensitivity hypothesis and accounts based on grandiose beliefs is the idea that illusions of control depend on the presence of signal-like noise, instead of a tendency to judge that one is in control irrespective of the evidence. This point can be demonstrated by analogy to perceptual decision making, where observers detect weak signals embedded in noise. In such tasks observers often falsely report that stimuli were present when faced with pure sensory noise (Green & Swets, 1966). Classic theories assumed that these ‘false alarms’ were strategic guesses (Swets et al., 1961). However this assumption has been questioned by recent work revealing that false alarms tend to occur when observers experience sensory noise that spuriously resembles the target (Wyart et al., 2012). Under these circumstances, a false alarm is completely rational, since observers have no way of distinguishing ‘true’ signals from signal-like noise (Green & Swets, 1966; Kloosterman et al., 2019; Wyart et al., 2012).

We suggest that illusions of control could arise in a conceptually similar fashion – without any grandiose biases, agents may experience feelings of agency when they detect objectively uncontrolled events that correlate with their actions due to random environmental fluctuations. This alternative explanation has been overlooked when theorising about the adaptive nature of illusions of control and their variation in different populations. As a proof-of-concept we conducted an experiment where participants made agency judgements about an observed cursor that could be spatiotemporally yoked to their actions (‘control’ trials) or follow an independent trajectory (‘no control’ trials). This task allowed us to dissociate objective sensitivity to agency from general biases to report control. We also applied reverse correlation techniques, calculating the spatiotemporal correlation between observed and executed movements on each trial and using this to predict the given response (‘agency’ or ‘no agency’). We examined whether sensitivity to these spurious correlations was sufficient to generate ‘illusions’ of control (i.e., biases) and simulated and modelled task performance to investigate whether unbiased agents may nonetheless exhibit illusions of control.

## 2 Method

### 2.1 Participants

The final sample for this study comprised 48 adults (27 women, 21 men; mean [SD] age 23 [4.5] years) with no psychiatric or neurological illness and normal or corrected vision. Two additional participants were tested but not analysed due to a technical malfunction (corrupted data files). Ethical approval was received from Birkbeck, University of London. Sample size was determined on the basis of pilot testing. A separate pilot experiment found illusions of control in agency judgements (*c* < 0) with an effect size of *d* = 1.02 (95% CI: 0.54–1.49). A sample of 48 participants therefore provides >90% power to detect effects at least as large as the lower bound of this interval.

### 2.2 Procedure

**Fig. 1:**
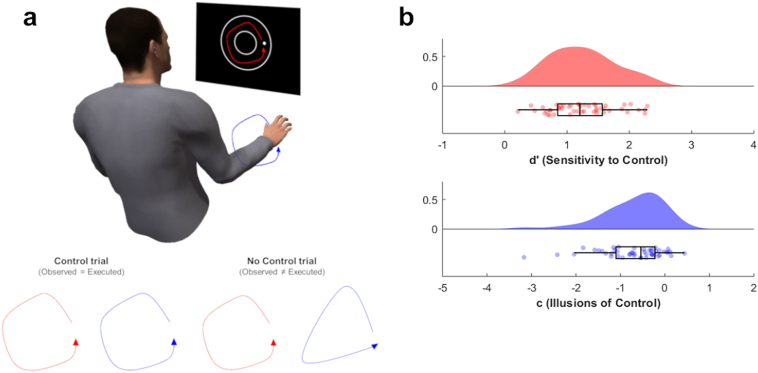
Motion tracking task and signal detection results: a) Participants performed counter-clockwise hand movements and observed similar movements onscreen. They were asked at the end of each trial whether their action controlled the trajectory of the observed dot. Sometimes these onscreen movements were entirely yoked to the participant's movements and were therefore controlled by them (‘control’ trials). Sometimes they were trajectories from previous trials and therefore participants did not control them (‘no control’ trials). b) Signal detection analyses revealed that participants were more likely to say they controlled the cursor movement on control trials relative to no control trials (sensitivity - *d*′), and were also more likely than not to report being in control regardless of trial type (bias - *c*; lower, negative values reflective of an illusion of control). Raincloud plots display probability density estimates (upper) and box and scatter plots (lower). Boxes denote lower, middle and upper quartiles, whiskers denote 1.5 interquartile range, and scattered dots denote individual participant datapoints (N = 48). Raincloud plots devised by Allen et al. (2019).

The task was run in MATLAB using Cogent 2000. Participants sat ~60 cm from a monitor, wearing a pair of glasses which selectively occluded view of their hands. Participants' hands were placed above an infrared motion tracker that recorded their actions. At the beginning of each trial participants viewed a white dot cursor that tracked the position of their palm and were required to move this dot into a start zone (demarcated by a wedge). After remaining in the start zone for 2000 ms, a ‘bagel’ shape appeared on screen. Participants were instructed to execute an anti-clockwise circular hand rotation (Fig. 1a). They were informed that on ‘control’ trials (50%) the dot would follow the trajectory of the participant's hand rotation, whereas on ‘no control’ trials (50%) the dot trajectory would be controlled by the computer, and were made aware that both trial types were equally likely. On ‘no control’ trials, the dot was animated with a movement trajectory from a previous trial, randomly-selected with replacement, once participants began to move. Stimulus presentation lasted for 2000 ms, and participants were trained to produce approximately one full rotation in this period. If the observed cursor moved outside the ‘bagel’ (regardless of trial type) participants received error feedback, judgements were not elicited and the trial was later repeated. This ensured that participants' agency judgements were not influenced by the success or failure of the intended movement (c.f. Oishi et al., 2018). Otherwise participants were presented with a question screen to judge whether they did or did not control the trajectory of the dot (responding with their left thumb). The main task comprised 300 trials in a randomised order, with breaks every 20 trials.

## 3 Results

### 3.1 Signal detection analyses: do agents experience illusions of control?

On each trial, the cursor was either yoked to the participant's action (‘control’ trials) or programmed to follow an independent trajectory from a previous trial (‘no control’ trials). Participants judged whether they controlled the trajectory of the observed dot. Standard signal detection theoretic measures (Green & Swets, 1966) of sensitivity (*d*′) and bias (*c*) were calculated from hit rates (proportion of ‘agency’ responses on control trials: mean [sd] = 0.88 [0.11]) and false alarm rates (proportion of ‘agency’ responses on no control trials: mean [SD] = 0.36 [0.21]). Broadly, *d*′ reflects the extent to which participants are more likely to report control on control trials than no control trials, and *c* measures whether they exhibit a generalised tendency to report control regardless of trial type (see Supplementary Methods for precise calculations). This analysis found that participants could discriminate between ‘control’ and ‘no control’ trials (mean *d*′ = 1.24, one sample *t*_47_ = 16.66, *p* < .001, *d* = 2.41; Fig. 1b), but also showed a bias to report agency regardless of trial type (mean *c* = −0.71, one sample *t*_47_ = 6.94, *p* < .001, *d* = 1.0; Fig. 1b). This latter finding suggests that our paradigm induces robust ‘illusions of control’, but unlike most previous work, using a paradigm that actually allows separation of sensitivity from bias.

### 3.2 Reverse correlation: are agents sensitive to spurious correlations?

**Fig. 2:**
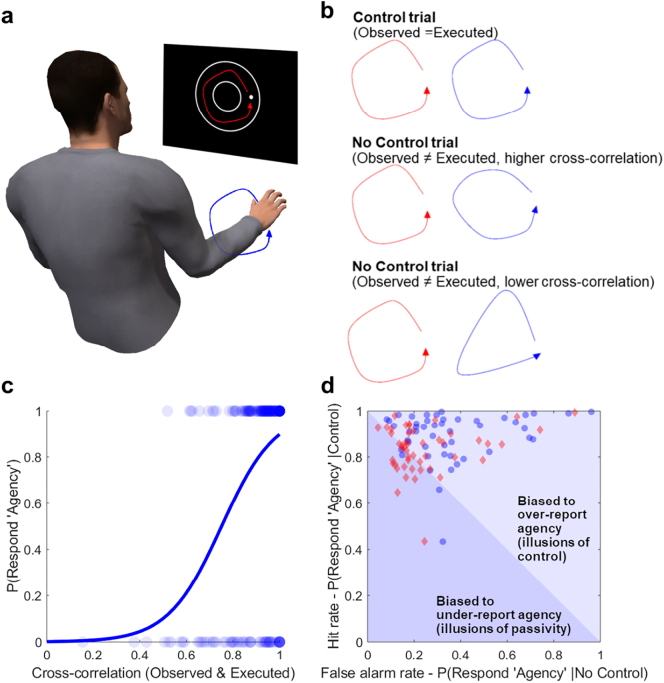
Calculating sensitivity to incidental correlations and simulating illusions: In this task (a) there are random fluctuations in the correspondence between action-outcomes on ‘no control’ trials that can be quantified by cross-correlation (b). Reverse correlation techniques found that participants were sensitive to these spurious correlations (c) and data simulated only from sensitivity to these correlations (red diamonds) recreated the ‘illusions of control’ seen on real trials (blue circles; both N = 48 d). (For interpretation of the references to color in this figure legend, the reader is referred to the web version of this article.)

We subsequently used reverse correlation (Wyart et al., 2012) to examine whether sensitivity to correlations could in fact account for bias as well as sensitivity effects. Specifically, signal detection analyses separate overarching biases from sensitivity by comparing the tendency to give a certain response (‘control’) regardless of trial type, and more readily for the control than no control trials, respectively. However, this blunt separation does not account for the fact that no control trials differ from each other in the extent to which they resemble control trials, and therefore both sensitivity and bias effects can in principle arise from signal sensitivity. For each trial we computed the spatiotemporal cross-correlation between the observed and executed motion trajectories. Logistic functions fit to each participant used trial-wise cross-correlation values to predict trial-wise responses (i.e. ‘agency’ or ‘no agency’; see Fig. 2).

The slopes of these functions (*β*_1_) reflect the weight given to correlations when making judgements. These values were positive (mean *β*_1_ = 19.58, *t*_47_ = 11.24, *p* < .001, *d* = 1.62), suggesting – perhaps unsurprisingly – that the likelihood of reporting agency increases as the random noise spuriously resembling the target increases. In other words, this value confirms that agents are sensitive to incidental correlations between actions and outcomes when making agency judgements. The constant of these functions (*β*_0_) captures any general bias to pick either response. A strong prediction of the grandiosity account – that agents are biased to overestimate control irrespective of objective facts – is that this parameter should be positive, indicating a general tendency towards agency regardless of facts. In fact, we found that these values were negative, suggesting that participants were not generally biased to report ‘agency’ regardless of correlations (mean *β*_0_ = −17.79, *t*_47_ = 10.38, *p* < .001, *d* = 1.50). It is worth noting that the same effects on *β*_1_ (*t*_47_ = 10.64, *p* < .001) and *β*_0_ values (*t*_47_ = 10.60, *p* < .001) were obtained for functions modelled only to ‘no control’ trials. However, we should perhaps not draw strong conclusions from this negative value, because correlations were typically positive (see Fig. 2c) and participants likely believed that they should often report no control. Under these task constraints a positive value may have been unlikely. We therefore incorporate modelling analyses in the following section that corroborate the proof-of-concept that in principle ‘illusions of control’ could arise purely through sensitivity to incidental correlations.

### 3.3 Computational modelling: does a sensitive agent experience illusions of control?

To confirm that sensitivity to correlations alone could generate illusions of control, we first used these modelled logistic functions to simulate detection performance for hypothetical trials. To this end we took 300 random pairs of observed and executed trajectories from each participant's real data and calculated the spatiotemporal cross-correlation that would have been obtained on these hypothetical trials. These values were fed into the participant's modelled function to simulate a false alarm rate (i.e. probability of responding ‘agency’). Hit rates were simulated similarly from a hypothetical trial where observed and executed movements were fully dependent (cross-correlation = 1). These simulated data (see Fig. 2d) showed the same tendency to report agency regardless of trial type (mean *c* = −0.22, one sample *t*_47_ = 2.54, *p* = .015, *d* = 0.366). Therefore, illusory experiences of control can be generated by underlying functions with no evidence-independent bias towards reporting agency.

**Fig. 3:**
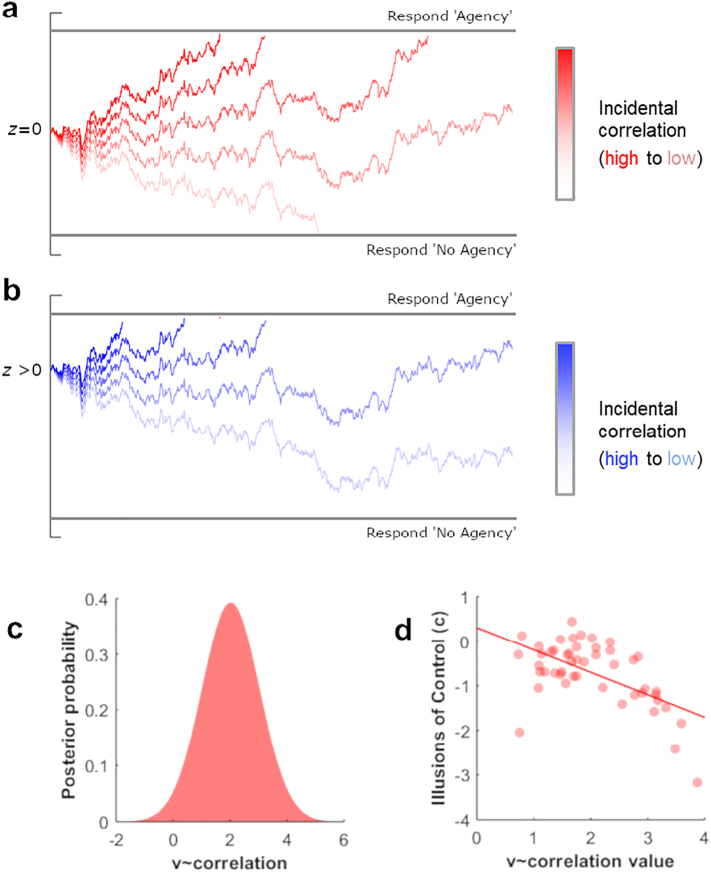
Computational modelling and links to illusions of control: a) An illustration of the ‘sensitive agent’ model, where the decision process is coupled to the incidental correlation experienced between actions and outcomes. b) An illustration of the ‘grandiose agent’ model, which is identical to the ‘sensitive agent’ apart from the fact that the start-point of the accumulation process can also be shifted towards one response or another. c) Posterior probability estimates for group-level parameter v ~ correlation. The v ~ correlation parameter, describes the coupling between decisions and incidental correlations between executed and observed motion trajectories, where positive values indicate a tendency to respond ‘agency’ when these correlations are higher. d) Relationship between subject-specific v ~ correlation values and empirical illusions of control (c values). Lower c values indicate stronger illusions of control.

Second, as already noted, we perhaps should not draw strong conclusions about the nature of the intercept effect in this paradigm. Other methods may provide a more powerful proof-of-concept that in principle ‘illusory’ control can arise purely through sensitivity to objective action-outcome relationships. To this end, we compared the sensitivity hypothesis and grandiosity hypothesis by modelling the agent's decision process under the drift diffusion model. Notably, the grandiosity hypothesis accommodates the fact that one is sensitive to correlations but assumes additional overarching biases that shift the likelihood of reporting control. Drift diffusion models conceptualise two-choice decisions (e.g. ‘am I in control?’) as a noisy process of evidence accumulation towards a decision boundary (Ratcliff et al., 2016). Evidence is sampled by decision units and when enough evidence has been accumulated to reach a decision boundary, the appropriate response is triggered. The evidence accumulation process is based upon units representing evidence, even if the physical events themselves have terminated by the start of the response window. We created two models – a *sensitive agent* model and a *grandiose agent* model. In the *sensitive agent* model (Fig. 3a) evidence accumulation (*v*) on ‘no control’ trials was determined by the incidental correlation between observed and executed motion trajectories. Under this model, agents would be more likely to respond ‘agency’ in the presence of stronger incidental correlations, and more likely to respond ‘no agency’ when correlations are weaker. According to the sensitivity hypothesis, the strength of this coupling between evidence accumulation and incidental correlations (*v* ~ correlation) determines whether an agent will experience illusions of control. The *grandiose agent* model (Fig. 3b) was identical to the sensitive agent but included an additional free-parameter – the start-point of the accumulation process (*z*). Changes in the start-point reflect general biases towards one kind of response irrespective of the evidence received. For example, under the grandiosity hypothesis one would expect agents to shift their start-point nearer to the ‘respond agency’ boundary, reflecting a general tendency to over-report one's control over external events, and shifts in this parameter would explain illusions of control. We fit both of these models to our data to determine whether ‘false alarms’ in our task were best described by a *sensitive agent* or *grandiose agent* model. Each model was specified and estimated using the hDDM package, which uses a hierarchical Bayesian modelling approach to estimate simultaneously group-level and subject-specific parameters (Wiecki et al., 2013).

We simulated data from the *sensitive agent* and *grandiose agent* models to determine how well these recovered patterns in our data. Simulations were conducted using the hDDM package, using the posterior distribution of parameter estimates to generate 3000 simulated decisions for each trial for each participant. For each trial, we calculated the proportion of simulated decisions where the ‘agency’ response was given, i.e., the probability of a false alarm. Averaging these trial-wise probabilities across all trials allowed us to compute a simulated false alarm rate for each participant. This procedure was performed separately for each model, yielding a simulated false alarm rate for the *sensitive agent* and *grandiose agent* model for each participant.

Inspecting the group-level parameter estimates for the *sensitive agent* model suggested that agents did indeed couple evidence accumulation to experienced correlations (mean *v* ~ correlation: 2.02). Moreover, we found a strong relationship between the weight an individual participant gave to incidental correlations and their experience of illusory control (*r*_48_ = −0.585, *p* < .001; see Fig. 3d). More importantly, using a stepwise linear regression, we determined how well these simulated false alarm rates captured true false alarm rates, and whether any additional variance was explained by the *grandiose agent* model. In the first step, we used simulated false alarm rates for the simpler *sensitive agent* model to predict empirical illusions of control (*c* values) experienced by each participant. This model was highly significant (*R*^2^ = 0.665, *F*_1,46_ = 91.37, *p* < .001), with false alarm rates from the *sensitive agent* strongly predicting the empirically obtained illusions. In the second step, we included simulated false alarm rates from the *grandiose agent* model as an additional predictor of empirical illusions of control. While the regression model remained highly significant (*R*^2^ = 0.669, *F*_2,45_ = 45.54, *p* < .001), this analysis found no significant improvement in model fit when this additional predictor was included (*F*_1, 45_ = 0.568, *p* = .455). This pattern suggests that the tendency to report ‘illusory control’ is well-explained by the simpler *sensitive agent* model, and that no additional variance in participant decisions is explained by the more complex *grandiose agent* model that incorporates evidence-independent biases.

## 4 Discussion

We found that agents experienced robust illusions of control – frequently reporting agency over events that they did not truly influence. However, reverse correlation analysis revealed that these judgements reflected sensitivity to incidental correlations between actions and outcomes, and simulations showed that this sensitivity – without any additional biases – was sufficient to generate illusions of control.

These results demonstrate that illusions of control may not imply grandiose beliefs about our capabilities that disregard objective evidence (Alloy & Abramson, 1979; Presson & Benassi, 2003). They in fact allow us to conceptualise illusions of control in line with other findings where we are highly sensitive to variations in the contingency between our actions and their outcomes (Dickinson et al., 1984; Elsner & Hommel, 2004; Moore et al., 2009; Moore & Haggard, 2008). They are also consistent with recent findings from perceptual decision making where biases to report seeing signals in noise can reflect spurious signals rather than complete hallucinations (Wyart et al., 2012). In other words, we find that an observer frequently experiences an ‘illusion’ that they controlled the environment when incidental correlations hold between what they do and what they see. These data suggest these experiences are ‘illusory’ in one sense but not in another – agents ascribe agency when there truly is none, but they do not necessarily make these subjective ascriptions without objective evidence.

The illusory claims are typically made based because individuals report control when events are not in fact caused by actions, e.g., when the occurrence of lightbulb flashes in an experiment is in fact determined randomly (Alloy & Abramson, 1979) or traffic lights are controlled by timers. The paradigms typically examine how often agency is reported, or implicit correlates of agency are found, when one was not in control. They do not separate sensitivity from bias. More importantly, the precise contingencies are not usually calculated and the experienced correlations are likely to be above zero. For example, ‘no control’ settings in laboratory paradigms usually present sensory events (e.g., lightbulb flashes) that are more frequent in the presence of action, even if the precise temporal relationship is modulated. Similarly, if we always press the placebo button at pre-programmed traffic lights, the lights will never change without the press. It is therefore critical to establish true action-outcome contingencies if wishing to make claims about the extent to which these contingencies do or do not determine one's sense of agency.

We have provided a proof-of-concept for how illusory control *could* result from sensitivity to objective relationships but future work must establish whether it always does and whether other factors influence these illusions. First, these effects may depend upon the proportion of control relative to no control trials. The magnitude of biases may rationally increase with the number of control trials, but equally could decrease if participants believe they should give a substantial number of ‘no control’ responses. Second, it has previously been argued that ‘illusions of control’ are higher when the probability of action is higher (Yarritu et al., 2014), and therefore that we may exhibit biases to perceive frequent actions as more related to random outcomes. It would be interesting to examine whether this bias results from higher incidental correlations when more actions are performed. Third, some illusions of control may appear to result even when no action outcomes are experienced. For instance, classic studies reported grandiose beliefs about winning a card game before outcomes are known (Langer, 1975). In principle these effects may be governed by past action-outcome contingencies, but they may instead suggest that grandiose assumptions do influence beliefs about control in certain circumstances. Regardless of the outcome of future work, the critical finding from our proof-of-concept study is that ‘illusions of control’ *can* arise purely through unbiased sensitivity to objective relationships – a logical point that appears to have been missed by some when theorising about their possible adaptive value – not that they necessarily always will. Indeed, one possibility to test in future work is that expectations may ‘tune’ agents to different kinds of evidence about control in the same way that expectations bias the gain we afford to the predicted sensory consequences of an action (Yon et al., in press).

These findings could prompt us to think differently about populations who display atypical illusions of control. For example, attenuated illusions of control in depression have prompted claims that these patients possess a ‘sadder but wiser’ view of their capabilities (Alloy & Abramson, 1979). However, this idea may need revisiting if illusions can reflect a high sensitivity to weak action-outcome relationships rather than a disregard for evidence. Specifically, ‘depressive realism’ could reflect a reduced sensitivity to weak correlations between actions and outcomes – consistent with evidence that depression is associated with difficulties in tracking contingencies between actions and rewards (Chase et al., 2010). The framework described here may also prove useful in determining whether other apparent biases in the sense of agency arise due to global biases in decision making, or changes in sensitivity to incidental correlations. For example, the experience of agency is relatively exaggerated for positive over negative outcomes (Takahata et al., 2012; Yoshie & Haggard, 2013). Such findings could reflect self-serving biases where agents take credit for good consequences. Alternatively, they could also arise if agents are more sensitive to incidental correlations between actions and positive outcomes (e.g. because they attend to them).

An important feature of judgements of control is that objective evidence ranges from perfect independence between action and outcome (correlation = 0) to perfect dependency (correlation = 1). By analogy to studies of perception, this means judgements of control are less like discriminations (e.g., is this line tilted left or right?) where observers can in principle process both positive and negative evidence for a particular outcome, and more like detection tasks (e.g., was there a line or not?). Intriguingly, observers in perceptual tasks tend to have poorer insight into their ability to judge absence over presence (Kanai et al., 2010; Meuwese et al., 2014), possibly because the former places particular demands on metacognitive self-monitoring mechanisms (Mazor et al., 2020). This asymmetry may have important implications for how agents actively monitor what they *cannot* control in certain kinds of environments (Wen et al., 2020).

A key question our data raise is whether high sensitivity to imperfect correlations makes our sense of agency more or less veridical in natural environments. This question is difficult to answer given the substantial variability in the absolute correlations we experience when we are truly in control. While there is a close to perfect correlation between our actions and the direct movement of our body, the influence we have on a temperamental friend or an uncooperative coffee machine may be considerably weaker. An important challenge will be to measure the correlations that occur between actions and different kinds of events in natural settings to assess whether human observers calibrate the weight they give to experienced correlations to this expected range. These types of measurement will be especially important in determining whether these biases are present in most healthy individuals because feeling in control in the face of imperfect correlations may be appropriate in many environments. Indeed, those who persevere with an unresponsive coffee machine or temperamental friend may often enjoy a fitness advantage but *not* because they are ‘unrealistically optimistic’ (Alloy & Abramson, 1979; Bandura, 2002; Haselton & Nettle, 2006; Johnson & Fowler, 2011; Presson & Benassi, 2003; Vázquez, 1987). Their actions may in fact influence the world but with an imperfect contingency. Expecting true agency to be associated with perfect action-outcome contingencies may indeed be maladaptive (e.g., depression), as previously assumed, but may in fact reflect a *less* wise, rather than wiser, appraisal of our ability to influence the world around us.

In conclusion, our results demonstrate that illusions of control can arise because agents are exquisitely sensitive to the relationships between actions and effects in an imperfect world and pick up on those that occur purely by chance.

## Data availability

Data are available as an online attachment to this manuscript.

## CRediT authorship contribution statement

All authors contributed to the design of the study. C.B. collected the data, which was analysed and interpreted by D.Y. in conjunction with all other authors. D.Y. wrote the manuscript and all authors were involved in revisions. C.P. supervised this work.

## Appendix A Supplementary data

Supplementary material 1 Image 1

Supplementary material 2 Image 2
