# Supplementary material for: Illusions of control without delusions of grandeur
Source: Cognition. 2020 Dec;205:104429. doi: 10.1016/j.cognition.2020.104429 (PMC7684464; doi:10.1016/j.cognition.2020.104429)
Supplement: Supplementary file 1 — Supplementary material 1 [file mmc1.docx]

**Illusions of control without delusions of grandeur – Supplementary Methods**

Daniel Yon^1,2*^, Carl Bunce^2^ and Clare Press^2^

1. ^Department of Psychology, Goldsmiths, University of London, UK^
2. ^Department of Psychological Sciences, Birkbeck, University of London, UK^

^*Corresponding author:^ [^d.yon@gold.ac.uk^](mailto:d.yon@gold.ac.uk)

1. *Signal detection analysis*

Standard signal detection theoretic measures of sensitivity and bias were calculated from each participant’s hit rate (HR) – P(Respond ‘Agency’| Control) – and false alarm rate (FAR) – P(Respond ‘Agency’ | No Control). Sensitivity was indexed by *d’*, where higher values indicate a superior ability to distinguish correctly between control and no control trials. *d’* is calculated as:

$$d^{'}= z\left( HR \right)-z(FAR)$$

Bias was indexed by *c,* where negative values indicate a bias to report agency on no control trials. *c* is calculated as:

$$c= -.5[z\left( HR \right)+z\left( FAR \right)]$$

1. *Spatiotemporal cross-correlation between observed and executed trajectories*

The spatiotemporal dependence between actions and events was determined by computing the mean-centred two-dimensional crosscorrelation coefficient between observed (*obs*) and executed (*exec*) motion trajectories. A crosscorrelation of 0 indicates that trajectories are perfectly independent, while 1 indicates a perfectly dependent relationship between observed and executed trajectories – as occurs on ‘control’ trials. This coefficient was calculated as:

$$r_{cross}= \frac{\sum_{m} \sum_{n} \left( {obs}_{mn}-\bar{obs} \right)\left( {exec}_{mn}-\bar{exec} \right)}{\sqrt{{(\sum_{m} \sum_{n} \left( {obs}_{mn}-\bar{obs} \right)}^{2}}{)(\sum_{m} \sum_{n} \left( {exec}_{mn}-\bar{exec} \right)}^{2})}$$

1. *Reverse correlation analysis*

We used a maximum-likelihood estimation procedure to fit logistic functions to participant behaviour, predicting binary agency judgements (‘agency’ or ‘no agency) based on the crosscorrelation between actions and outcomes on a given trial (r_cross_). This function had the form:

$$P\left( Respond{}^{'}Agency^{'} \right)= \frac{1}{1+exp(-(\beta_{0}+ \beta_{1}.r_{cross})}$$

In this function two free parameters are estimated: - *β_0_* and *β_1_*. *β_0_* controls the constant on the function, capturing any tendency toward reporting being in control that is independent of the experienced correlations (positive values = general biases to report agency). *β_1_* controls the slope of the function, and reflects the weight agents give to experienced correlations when making their decisions (positive values indicate stronger weightings).

1. *Simulating ‘illusions of control’*

Hypothetical task performance was simulated for each participant using the parameters modelled in the reverse correlation analysis. This involved creating 300 random pairs of observed and execution trajectories as dummy ‘trials’. The crosscorrelation between observed and executed trajectories was calculated for each ‘trial’ as for the real data (see above), and these values were fed to the modelled parameters modelled during reverse correlation. This yields a P(Respond ‘Agency’) for every trial, which can be averaged to form a simulated false alarm rate. A hit rate can be simulated in a similar way by determining the P(Respond ‘Agency’) yielded when crosscorrelations are perfect – as they are on ‘control’ trials in the main experiment.

Simulated hit rates and false alarm rates were used to calculate signal detection theoretic measures of sensitivity (*d’*) and bias (*c*) as for the real data (see above).

1. *Hierarchical drift diffusion modelling*

To further compare the sensitivity and grandiosity hypotheses as explanations for illusions of control, we modelled participant choices and reaction time using the drift diffusion model (DDM).

We fit two models to participant data. The first was a *sensitive agent* model, where the rate of evidence accumulation (*v*) was controlled by the experienced correlation between observed and executed motion trajectories. When this coupling is positive (i.e. v~correlation >0), agents are more likely to respond ‘agency’ when experienced correlations are higher, and more likely to respond ‘no agency’ when experienced correlations are lower. The second was a *grandiose agent* model, which was identical to the sensitive agent but included an additional free-parameter – the start-point of accumulation (*z*). When this start-point parameter is positive, agents are biased to respond ‘agency’ irrespective of the evidence they sample on a given trial.

Models were fit to participant data using the hierarchical drift diffusion model (hDDM) package in Python (Wiecki, Sofer, & Frank, 2013), which uses Monte Carlo Markov Chain (MCMC) sampling to simultaneously estimate group-level and subject-specific model parameters. Each model was estimated with MCMC sampling with 30,000 samples (‘burn-in’ = 7500), and model convergence was assessed by inspecting chain posteriors.

**Supplementary references**

Wiecki, T. V., Sofer, I., & Frank, M. J. (2013). HDDM: Hierarchical Bayesian estimation of the Drift-Diffusion Model in Python. *Frontiers in Neuroinformatics*, *7*, 14. https://doi.org/10.3389/fninf.2013.00014
